# Target product profile for a dengue pre-vaccination screening test

**DOI:** 10.1371/journal.pntd.0009557

**Published:** 2021-07-29

**Authors:** Noah Fongwen, Annelise Wilder-Smith, Duane J. Gubler, Eng Eong Ooi, Edsel Maurice T. Salvana, Xavier de Lamballerie, Piero L. Olliaro, Rosanna W. Peeling

**Affiliations:** 1 International Diagnostics Centre, Department of Clinical Research, London School of Hygiene and Tropical Medicine, London, United Kingdom; 2 Heidelberg Institute of Global Health, University of Heidelberg, Germany; 3 Institute of Social and Preventive Medicine, University of Bern, Switzerland; 4 Programme in Emerging Infectious Diseases, Duke-NUS Medical School, Singapore; 5 Institute of Molecular Biology and Biotechnology, National Institutes of Health, University of the Philippines Manila, Manila, Philippines; 6 Unité des Virus Emergents (UVE), Aix Marseille Université, IRD 190, INSERM 1207, IHU Méditerranée Infection, Marseille, France; 7 Centre for Tropical Medicine and Global Health, University of Oxford, Oxford, United Kingdom; University of Peradeniya Faculty of Medicine, SRI LANKA

## Abstract

With increasing geographic spread, frequency, and magnitude of outbreaks, dengue continues to pose a major public health threat worldwide. Dengvaxia, a dengue live-attenuated tetravalent vaccine, was licensed in 2015, but post hoc analyses of long-term data showed serostatus-dependent vaccine performance with an excess risk of hospitalized and severe dengue in seronegative vaccine recipients. The World Health Organization (WHO) recommended that only persons with evidence of past dengue infection should receive the vaccine. A test for pre-vaccination screening for dengue serostatus is needed. To develop the target product profile (TPP) for a dengue pre-vaccination screening test, face-to-face consultative meetings were organized with follow-up regional consultations. A technical working group was formed to develop consensus on a reference test against which candidate pre-vaccination screening tests could be compared. The group also reviewed current diagnostic landscape and the need to accelerate the evaluation, regulatory approval, and policy development of tests that can identify seropositive individuals and maximize public health impact of vaccination while avoiding the risk of hospitalization in dengue-naive individuals. Pre-vaccination screening strategies will benefit from rapid diagnostic tests (RDTs) that are affordable, sensitive, and specific and can be used at the point of care (POC). The TPP described the minimum and ideal characteristics of a dengue pre-vaccination screening RDT with an emphasis on high specificity. The group also made suggestions for accelerating access to these RDTs through streamlining regulatory approval and policy development. Risk and benefit based on what can be achieved with RDTs meeting minimal and optimal characteristics in the TPP across a range of seroprevalences were defined. The final choice of RDTs in each country will depend on the performance of the RDT, dengue seroprevalence in the target population, tolerance of risk, and cost-effectiveness.

## Author summary

This paper describes the consensus on the minimum and ideal performance and operational characteristics of rapid tests that would be used for dengue pre-vaccination screening. This profile will incentivize industry to develop better pre-vaccination screening tests. The choice of which test to use depends on the seroprevalence of the population targeted for vaccination and the optimal balance between benefit and risks. The group also made suggestions for accelerating access to these pre-vaccination screening tests through streamlining regulatory approval and policy development.

## Introduction

Dengue is a major public health problem with more than 3.6 billion people at risk for dengue virus (DENV) infection and an estimated 390 million infections annually in over 120 tropical and subtropical countries [1,2]. With increasing geographic spread, frequency, and magnitude of outbreaks, dengue has also become a major problem in international travelers [3,4]. In the absence of truly effective and sustainable vector control measures, a dengue vaccine is urgently needed. The first dengue vaccine was licensed in 2015: the live-attenuated recombinant tetravalent vaccine CYD-TDV (Dengvaxia) developed by Sanofi Pasteur. However, post hoc analyses of the long-term data in the multicountry Phase III trials showed serostatus-dependent vaccine performance of Dengvaxia. An excess risk of hospitalized and severe dengue was found in year 3 after vaccination in baseline seronegative vaccine recipients, while in seropositive vaccine recipients, the vaccine was efficacious and safe [5]. The World Health Organization (WHO) recommended that only persons with evidence of a past DENV infection (seropositive) should receive the vaccine; hence, pre-vaccination screening for dengue serostatus is needed [6]. To support the strategy, WHO and other expert panels highlighted the urgent need for rapid diagnostic tests (RDTs) to determine serostatus. Pre-vaccination screening strategies will benefit from RDTs that are affordable, sensitive, and specific and can be used at the point of care (POC) in a population-wide program [6]. To date, no RDT has been licensed for the indication of determining dengue serostatus.

In this paper, we discuss the processes that led to the final target product profile (TPP) for a dengue RDT for pre-vaccination screening, development of RDTs in comparison to dengue ELISA testing, current RDT landscape and hurdles for marketing new RDTs, and considerations in RDTs performance to maximize public health impact.

### The processes toward TPP development

To develop the TPP for a dengue pre-vaccination screening RDT, face-to-face consultative meetings were organized by the Partnership for Dengue Control and the Global Dengue and *Aedes*-transmitted Diseases Consortium (GDAC) with follow-up regional consultations. The first face-to-face consultative meeting was in January 2019. Prior to the meeting, a preliminary draft of the TPPs was prepared based on online consultations and discussions with key regional experts. During the 2019 meeting, the preliminary draft was presented for further refinement through focus groups and individual discussions. Semi-structured interviews were also conducted with 16 different experts and country representatives from Latin America and Asia Pacific regions. A draft TPP was published as part of the 2019 meeting report [7]. A second face-to-face meeting was organized in January 2020. During this follow-up meeting, 10 more key informant interviews were conducted with country representatives and key opinion leaders. The International Diagnostics Centre (IDC) at the London School of Hygiene and Tropical Medicine (LSHTM) was mandated to lead the next steps toward finalizing the TPP. A technical working group was formed with the responsibility of developing consensus on a reference test against which candidate dengue pre-vaccination screening RDTs could be compared.

A meeting of the technical working group was convened online on May 14, 2020, with the goal of arriving at a consensus on the reference standard for the pre-vaccination screening test TPP. During the meeting, data were presented from comprehensive analyses of baseline samples from over 3,800 participants in the immunogenicity subsets of the CYD-TDV vaccine Phase III trials (CYD14 and CYD15). The updates provided the rationale and evidence supporting the selection of an appropriate reference standard.

### The reference standard and final TPP

To arrive at a reference standard, a comprehensive analysis of the advantages and disadvantages of different potential reference tests was performed based on data from the Phase III clinical trial of the CYD14 and CYD15 immunogenicity subset [8,9].

Plaque Reduction Neutralization Test 90 (PRNT_90_) is the most specific DENV serological test and is recommended by WHO for determining past dengue exposure in endemic areas [10]. However, neutralizing antibodies are only a small subset of antibodies produced in response to infection. Hence if PRNT is used as a reference standard alone, there will be false-negative pre-vaccination screening results that lead to people with prior dengue infection being denied vaccination. Therefore, some modifications should be made to minimize this potential bias. The nonstructural protein 1 (NS1) immunoglobulin G (IgG) ELISA and Plaque Reduction Neutralization Test 50 (PRNT_50_) can be used to minimize this bias. The dengue NS1 IgG ELISA assay offers excellent discrimination of previous dengue infection and shows no evidence of cross-reactivity with Japanese encephalitis and yellow fever, while results from a very limited number of post-Zika virus (ZIKV) and West Nile virus samples were inconclusive [11].

**Fig 1:**
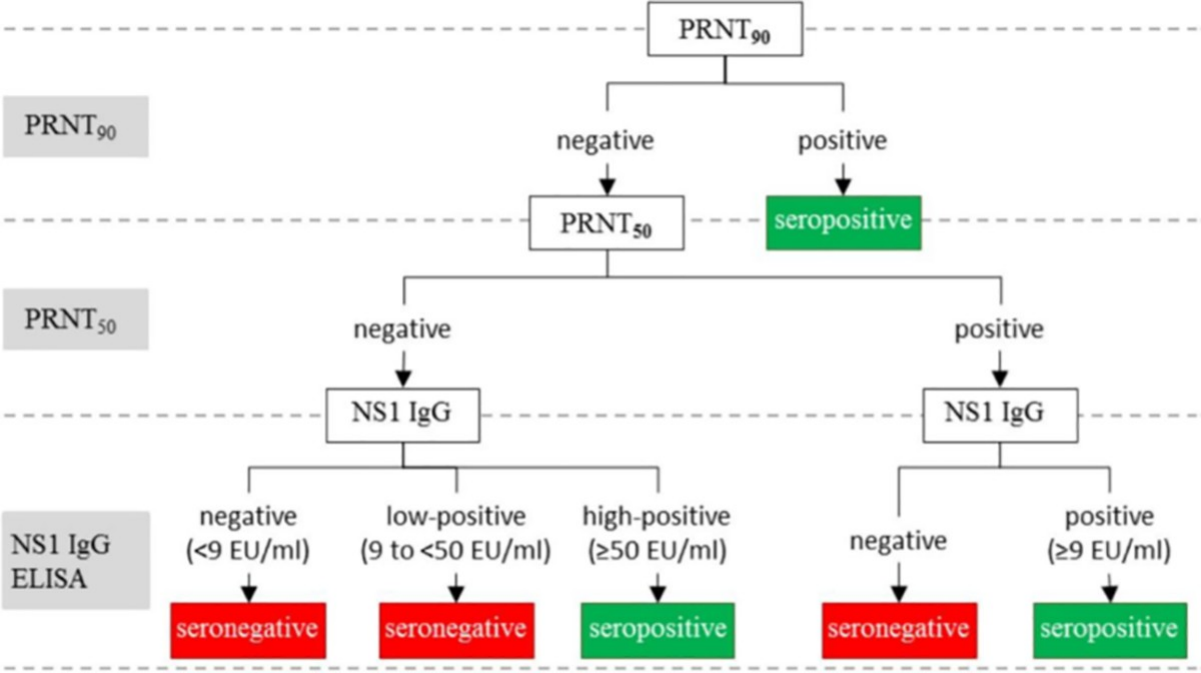
Algorithm for using PRNT90, PRNT50, and dengue NS1 IgG ELISA for reference dengue serostatus determination [12]. IgG, immunoglobulin G; EU/ml, ELISA Units per milliliter; NS1, nonstructural protein 1; PRNT_50_, Plaque Reduction Neutralization Test 50; PRNT_90_, Plaque Reduction Neutralization Test 90.

The technical working group considered the use of PRNT_90_, PRNT_50_, and dengue NS1 IgG ELISA as a reference dengue serostatus algorithm in Fig 1 [12].

The advantage of the above algorithm is that it may provide the most accurate representation of true dengue serostatus. However, the disadvantages are that PRNT requires specialized laboratory setting with assay experience. It is time consuming, requires relatively large serum volumes, and throughput is limited. Interlaboratory variability in PRNT assay methods may impact results. Dengue NS1 IgG ELISA is yet to be set up outside of research development sites.

The technical working group also considered selecting commercially available DENV IgG ELISAs that have performance characteristics close to this composite reference standard, but are widely available and can be performed in most laboratories. Sanofi Pasteur has published data showing that the Panbio Indirect and Euroimmun IgG ELISAs have the best performance profiles against the PRNT_90_, PRNT_50_, and NSI IgG as a serostatus reference standard [13]. The Euroimmun IgG ELISA exhibits a lower overall cross-reactivity to other flaviviruses, while the PanBio exhibits moderate levels of cross-reactivity to ZIKV and West Nile virus. This limits the use of the Panbio Indirect ELISA in areas with high ZIKV prevalence and a moderate sensitivity in detection of DENV serotype 4 monotypic immunes (56%). However, it was suggested that epidemiologically, as ZIKV emerged in dengue endemic areas, transmitted by the same vector, the prevalence of ZIKV seropositivity generally coincides with that of DENV; in other words, the prevalence of individuals positive to ZIKV and naive to DENV is probably very low.

Sanofi Pasteur further evaluated the performance of 3 IgG RDTs using PRNT_90_, the Panbio IgG, or the Euroimmun IgG ELISA as comparators, using baseline sera from 6 to 16 year olds in the CYD14/CYD15 immunosubsets. The results show that the PRNT_90_ as a comparator exhibits advantages over 2 commercial IgG ELISAs. Performance estimates for RDTs over a spectrum of sensitivities show that PRNT_90_ as comparator yields estimates that are closest to those with the comparator algorithm shown above. The IgG ELISAs overestimate sensitivity and underestimate specificity. These differences are accentuated for the high sensitivity IgG RDTs.

The technical working group concluded that, given the importance of using a test of high specificity for pre-vaccination screening, PRNT_90_ should remain as the comparator for the evaluation of pre-vaccination screening test. This is now shown in the final TPP (Table 1). The group also recommend that a reference panel be made available for the evaluation of pre-vaccination RDTs as PRNT assays are not widely available worldwide. Furthermore, the group recommend the development of an external quality assessment (EQA) program for pre-vaccination screening IgG RDTs and to check lot-to-lot variations.

**Table 1:** TPP for a dengue test for pre-vaccination screening.

Characteristic	Minimal	Optimal	Comments
Scope			
Goal of test	RDT for detection of dengue-specific IgG antibodies indicative of previous dengue infection		Detection of all 4 serotypes
Target population	Individuals eligible for dengue vaccination		Vaccine licensed for 9–45 year olds living in endemic areas
Target user	Minimally trained community health worker		Could be the same person who is giving the vaccine
Target use setting	Community-based settings (schools and community vaccination campaign), clinics, and hospitals		Should be usable in low to high endemicity settings
Healthcare system requirements	Functioning vaccination program with clear understanding and ability to communicate the risks and benefits of vaccination	Same as minimal, plus the following: - serosurveys - risk/benefit analysis - reference laboratory	
Assay characteristics			
Specimen type	Finger-prick whole blood ≤100 μl	Finger-prick whole blood ≤25 μl	
Specimen handling	Maximum 2 handling steps after finger-prick	Direct application of whole blood without handling	
Time to result	30 minutes	15 minutes	
Result interpretation	Visual/qualitative	Automated reader/semiquantitative grading of strength of positivity	
Price per test	≤$7.50 USD	≤$2.50 USD	
Biosafety/waste disposal	Simple waste biosafety disposal		
Assay stability: transportation	No cold chain	No cold chain, withstand transport stress	Use of vaccination supply chains may facilitate transportation of test kits
Assay stability: operating conditions and shelf life	10–30°C and 80% relative humidity, ≥12-month shelf life	5–40°C and 95% relative humidity or individually sealed tests with desiccants to enable humidity-proof packaging, ≥18-month shelf life	
Internal control	Internal process control line visually to indicate proper functioning	Presence of additional detection lines to identify cocirculating flavivirus antibodies for flow-type test formats, for example	Future research may demonstrate if other flavivirus antibodies will affect the dengue vaccine performance
Resulting reporting and assay connectivity	No connectivity; manual result reporting in vaccination record	Automated reader with connectivity for transfer of results to electronic medical records/databases and patient result notification	Adequate result reporting can also facilitate repeat testing of negative individuals
Test performance			
Clinical sensitivity	≥85%	≥95%	Specificity is a higher priority than sensitivity Performance shall be determined in appropriate samples Dengue seroprevalence will impact the required specificity of the test
Clinical specificity	≥95%	≥98%	
Reference standard	PRNT_90_		The PRNT_90_ assay was selected as the reference standard as it has the highest specificity
PPVs and NPVs	≥90%	≥95%	
Cross-reactivity	No cross-reactivity to other flaviviruses No cross-reactivity to circulating antibodies from other flavivirus vaccinations No cross-reactivity to endogenous substances and other pathogens		
Characterization of reference samples	Samples from individuals with the following: - proven past dengue infection - no known flavivirus exposure and no evidence of dengue IgG - proven previous infection with other flaviviruses - prior flavivirus vaccination	Samples from a well-characterized cohort including individuals with the following:virological confirmation of acute dengue infection with varying time points after resolution of acute infection - no known flavivirus exposure and no evidence of dengue IgG - proven asymptomatic past dengue infection - previous infection by other flaviviruses with varying time points after resolution of infection - previous infection by both dengue and another flavivirus with varying time points after resolution of infections - who have received other flavivirus vaccinations	

IgG, immunoglobulin G; NPV, negative predictive value; PPV, positive predictive value; PRNT_90_, Plaque Reduction Neutralization Test 90; RDT, rapid diagnostic test; TPP, target product profile.

### Current landscape of rapid diagnostic tests

WHO has called for the development of POC tests with adequate performance characteristics to identify prior DENV infection, i.e., high specificity and sensitivity in order to minimize vaccine risk and maximize individual and public health benefits. Until tests specifically designed for that purpose become available, WHO considered the use of IgG ELISAs and IgG-containing RDTs as temporizing tools depending on the epidemiological setting [6].

RDTs had variable sensitivities (40% to 70%) that were lower than those of the ELISAs (>/ = 90%). Cross-reactivity to other flaviviruses was low with RDTs (</ = 7%) but was more significant with ELISAs (up to 51% for West Nile virus and 34% for ZIKV). For each test, sensitivity appeared similar in samples from individuals with recent (<13 months) versus remote (3 to 4 years) virologically confirmed DENV infections. In general, dengue IgG RDTs were found to be more specific and less cross-reactive than ELISAs [13].

Some diagnostic developers have made progress in developing RDTs that can potentially be used for pre-vaccination screening. Sanofi Pasteur has codeveloped a dengue pre-vaccination screening IgG RDT that prioritizes very high specificity (to minimize the risk of vaccination of false-positive individuals), minimal to no flavivirus cross-reactivity, and high sensitivity to ensure detection of a high proportion of true dengue-seropositive individuals [9,13]. At the 2020 meeting, 4 diagnostic companies presented on the status of development of DENV IgG RDTs. These companies were Bio-Rad, BluSense, Chembio, and CTK Biotech. In general, the developers reported candidate assays of high specificities with some compromise on their sensitivities. All assays are easy to use with whole blood, serum, and plasma, but each assay has its own unique advantages and disadvantages. The Chembio assay is a multiplex lateral flow assay for DENV, ZIKV, and chikungunya, with quantitative detection and data connectivity using a digital reader. The BluSense immunomagnetic assay has connectivity capabilities and quantitative detection. The CTK Biotech assay is easy to use and has a long shelf life. The Bio-Rad assay is an easy-to-use lateral flow assay.

### Barriers in adoption of new diagnostic tests

Bringing new diagnostic tests to the market may take on average more than 10 years. There are 3 valleys of death that may limit the access of diagnostics. These include regulatory, policy, financial and health systems barriers. Regulatory barriers can be a major hurdle in ensuring access to quality-assured diagnostics, as often regulatory science has not kept pace with technological innovation.

**Fig 2:**
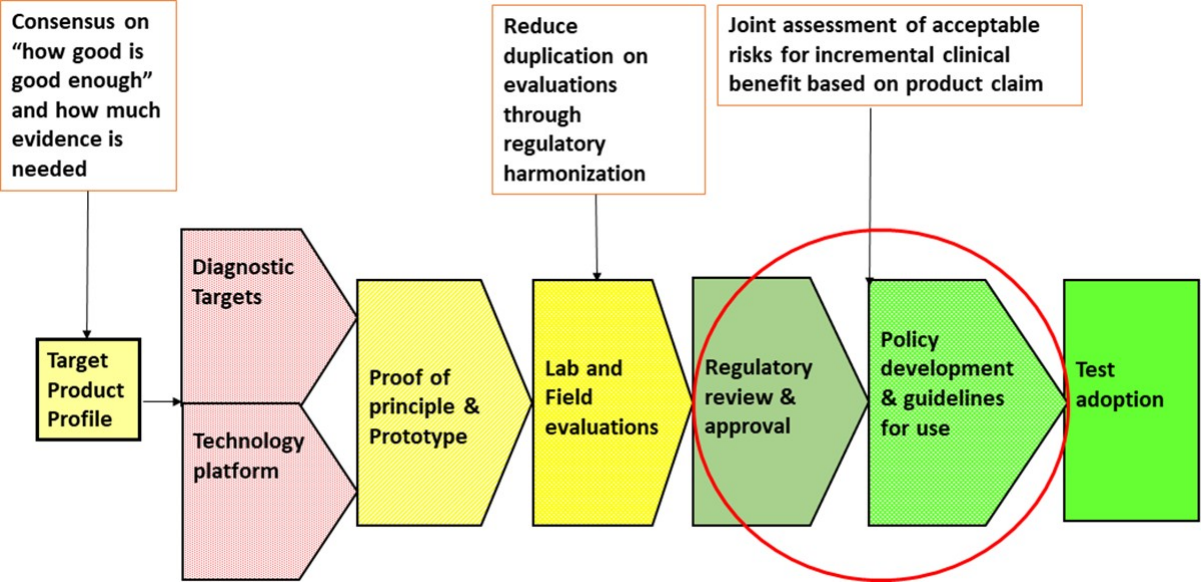
Proposed new regulatory policy framework to accelerate regulatory approval for IVD. IVD, in vitro diagnostics.

The paradigm of non-inferiority can no longer be used for the regulatory approval of accessible diagnostics. There is an urgent need for joint assessment of risks and benefits by regulators, policy makers, and subject matter experts to accelerate the access pathway. Successes in implementation of new diagnostics depend on engaging policy makers early in determination of test performance in settings and populations to maximize individual and public health benefits. Fig 2 illustrates this new regulatory framework that has been proposed as a critical step in reducing regulatory bottlenecks.

### Trade-offs in RDT performance, cost-effectiveness studies, and public health impact

Pre-vaccination RDTs are designed to identify the population eligible for vaccination. These tests should have very high specificity to exclude those individuals not eligible for vaccination (dengue seronegatives) to reduce potential harm by inadvertently vaccinating false-seropositive individuals. High specificity typically comes at the cost of test sensitivity, and, hence, a loss in detecting those previously exposed to dengue and who are the most likely to benefit from vaccination. Different modeling approaches show that in settings with high endemicity (prevalence >70%), this trade-off will result in little net change in vaccination impact compared to vaccination without prior screening. However, in settings with lower dengue transmission, the screen and vaccinate strategy would improve the impact of vaccination versus a no testing strategy. Positive predictive values (PPVs) and negative predictive values (NPVs), which combine positive and negative pretest probability and performance characteristics of a given test, have been proposed as alternative and more meaningful cross-setting indicators. While PPV constrains RDT accuracy in low prevalence settings, NPV constraints do so for high prevalence settings [14].

To fulfill both criteria, a very sensitive (>85%) and highly specific (>95%) RDT is required. According to our consensus TPP, a pre-vaccination screening RDT should be used in a seroprevalence setting at which its PPV is 90% or greater (Table 1). Countries should decide, based on their own seroprevalence levels and risk management approach, which levels of test performance and predictive values they would adopt. Table 2 presents a series of case scenarios of dengue seroprevalence ranges covered by tests meeting minimal and optimal criteria for performance (sensitivity and specificity) and predictive values. For instance, a test with 95% sensitivity and 98% specificity covers the 30% to 70% seroprevalence range with 95% PPV and 90% NPV. PPV and NPV need not be symmetrical, depending on a country’s situation and choices. For example, a test with 75% sensitivity works just as well as a test with 80% sensitivity for populations with seroprevalences between 25% and 30%. However, as shown in Table 2, at a seroprevalence of 50%, a test with sensitivities of 75% and 80% will lead to NPVs of 81% and 83%, respectively. Increasing test sensitivity to 90% will restore the PPV and NPV to acceptable levels, i.e., above 90%.

**Table 2:** Relationship between test performance and its predictive values when screening populations of different seroprevalence.

Dengue seroprevalence	Test performance		Predictive values		Distribution of test outcomes among 10,000 people screened				Mitigation
	Se	Sp	PPV	NPV	True positive Vaccine benefit	False positive Vaccine risk	True negative Not vaccinated	False negative Denied vaccine	
**15%**	75%	98%	**87%**	96%	1,125	170	8,330	375	**Confirm positives**
	80%	98%	**88%**	97%	1,200	170	8,330	300	
	90%	98%	**89%**	98%	1,350	170	8,330	150	
	95%	98%	**89%**	99%	1,425	170	8,330	75	
	75%	99%	93%	96%	1,125	85	8,415	375	
**25%**	75%	98%	93%	92%	1,875	150	7,350	625	
	80%	98%	93%	94%	2,000	150	7,350	500	
	90%	98%	94%	97%	2,250	150	7,350	250	
	95%	98%	94%	98%	2,375	150	7,350	125	
**50%**	75%	98%	97%	**80%**	3,750	100	4,900	1,250	**Confirm negatives**
	80%	98%	98%	**83%**	4,000	100	4,900	1,000	
	90%	98%	98%	91%	4,500	100	4,900	500	
	95%	98%	98%	95%	4,750	100	4,900	250	

NPV, negative predictive value; PPV, positive predictive value; Se, sensitivity; Sp, specificity.

For settings with seroprevalences less than 16%, it may be useful to consider the use of a test with 99% specificity or use a 2-test algorithm to increase specificity. In low and moderately endemic settings, a screen-and-vaccinate strategy would streamline the use of vaccine, reduce the safety risk of vaccinating individuals without prior exposure to the virus, and drastically reduce the number of doses used against the additional expenses from testing a whole birth cohort. Cost-effectiveness is likely most sensitive to the specificity of the test, as a lack thereof will result in additional vaccine costs that are used to generate a net negative health impact through the vaccination of seronegative individuals. Published models diverge on their prediction of cost-effectiveness of a test and vaccinate strategy, spanning from not cost-effective to highly cost-effective for endemic countries including the Philippines and Brazil [9,15]. The assumed case fatality ratios may be a key driver for such differences. At the same time, cost-effectiveness models tend to underestimate the loss of effectiveness in terms of public health impact of vaccination campaigns when the test is not sensitive enough and would turn in false-negative results and miss individuals who would benefit from vaccination and help reduce transmission.

Modeling studies found that repeat testing could improve return on investment (ROI) despite increasing intervention costs. Thus, more detailed analyses should address questions on repeat testing and testing periodicity, in addition to real test sensitivity and specificity [15,16]. Our results follow from a mathematical model relating ROI to epidemiology, intervention strategy, and costs for testing, vaccination, and dengue infections. The authors applied this model to a range of strategies, costs and epidemiological settings pertinent to CYD-TDV, including a range of seroprevalences from 30% to 70% and vaccination both with and without an RDT (85% sensitivity and 95% specificity). Modeling indicates that it is possible to reduce hospitalization in the age-eligible cohorts by at least 15% and that from a societal perspective, it may be at least cost-effective to do so (under incremental cost-effectiveness ratios and ROI). This cost-effectiveness remains when considering multiple testing, and the use of a web-based app developed at LSHTM proved to aid public health officials in assessing whether an annual testing program is cost-effective based on the relative cost of the test and vaccine to the cost of a secondary infection (https://samclifford.shinyapps.io/Denvax_demo/).

## Discussion

The current dengue vaccine cannot be deployed without a concurrent prescreening strategy. While there are now new data on the dengue vaccine and some advancements toward the development of an RDT that can be used for pre-vaccination screening, current RDTs have high specificity but at the expense of a lower sensitivity. Future RDTs specifically designed for dengue pre-vaccination screening are yet to be developed, independently evaluated, undergo regulatory approval, and registered for use in countries.

In this paper, we provide indications as to what can be achieved with RDTs meeting minimal and optimal characteristics, but the choice of which levels of performance are acceptable will depend on a country’s appreciation of needs and tolerance of risks.

WHO, in 2019, put in place a Diagnostic Technical Advisory Group (DTAG) to facilitate the development of new TPPs [17]. In line with the 2021 priorities and with support from partners, the DTAG has already developed TPPs for onchocerciasis, lymphatic filariasis, schistosomiasis, soil-transmitted helminthiasis, human African trypanosomiasis, and leprosy. The TPPs for scabies, yaws, and mycetoma are near completion. Even though the dengue TPP development was not captured as a priority of the DTAG for this year, the work described here can serve as our collective contribution to WHO DTAG process and potentially expedite the evaluation and deployment of a test that is urgently needed for the deployment of dengue vaccines. The TPP process described in this paper is in line with WHO TPP development process and will be sent to the DTAG for their review and possible incorporation into WHO Research and Development (R&D) pathway when appropriate.

In the meantime, the Partnership for Dengue Control and GDAC will continue this work in collaboration with country partners. The next steps are to develop a mechanism and a protocol for the independent evaluation of candidate RDTs to be used for dengue pre-vaccination. A call for expression of interest to companies will be sent out, and sites in the IDC/LSHTM biobanking/evaluation network will be approached regarding their interest in participating in the independent evaluation. Most of these sites were part of WHO/TDR dengue and European Union–funded ZikaPLAN evaluation networks and are familiar with what needs to be done and have template agreements that they can sign with companies [18,19]. To accelerate access to pre-vaccination screening RDTs, IDC will work with regulators and policy makers to streamline the regulatory approval and policy development for these dengue screening tests through joint data review and assessment of acceptable risks for the incremental benefits of the vaccine for the population. This process will be initiated in parallel with the independent evaluations of the tests.

## References

[1] Bhatt S, Gething PW, Brady OJ, Messina JP, Farlow AW, Moyes CL, et al. The global distribution and burden of dengue. Nature. 2013;496(7446):504–7. doi: 10.1038/nature12060 23563266 PMC3651993

[2] Wilder-Smith A, Ooi EE, Horstick O, Wills B. Dengue. Lancet. 2019;393(10169):350–63. doi: 10.1016/S0140-6736(18)32560-1 30696575

[3] Redondo-Bravo L, Ruiz-Huerta C, Gomez-Barroso D, Sierra-Moros MJ, Benito A, Herrador Z. Imported dengue in Spain: a nationwide analysis with predictive time series analyses. J Travel Med. 2019;26(8). doi: 10.1093/jtm/taz072 31608405 PMC6927315

[4] Halstead S, Wilder-Smith A. Severe dengue in travellers: pathogenesis, risk and clinical management. J Travel Med. 2019;26(7):1–15. doi: 10.1093/jtm/taz062 31423536

[5] Sridhar S, Luedtke A, Langevin E, Zhu M, Bonaparte M, Machabert T, et al. Effect of dengue serostatus on dengue vaccine safety and efficacy. N Engl J Med. 2018;379(4):327–40. doi: 10.1056/NEJMoa1800820 29897841

[6] Wilder-Smith A, Hombach J, Ferguson N, Selgelid M, O’Brien K, Vannice K, et al. Deliberations of the Strategic Advisory Group of Experts on Immunization on the use of CYD-TDV dengue vaccine. Lancet Infect Dis. 2019;19(1):e31–8. doi: 10.1016/S1473-3099(18)30494-8 30195995

[7] Wilder-Smith A, Smith PG, Luo R, Kelly-Cirino C, Curry D, Larson H, et al. Pre-vaccination screening strategies for the use of the CYD-TDV dengue vaccine: A meeting report. Vaccine. 2019;37(36):5137–46. doi: 10.1016/j.vaccine.2019.07.016 31377079

[8] España G, Yao Y, Anderson KB, Fitzpatrick MC, Smith DL, Morrison AC, et al. Model-based assessment of public health impact and cost-effectiveness of dengue vaccination following screening for prior exposure. PLoS Negl Trop Dis. 2019;13(7):1–21. doi: 10.1371/journal.pntd.0007482 31260441 PMC6625736

[9] Bonaparte M, Zheng L, Garg S, Guy B, Lustig Y, Schwartz E, et al. Evaluation of rapid diagnostic tests and conventional enzyme-linked immunosorbent assays to determine prior dengue infection. J Travel Med. 2019 Dec;26(8):1–11. doi: 10.1093/jtm/taz078 31616949

[10] Product TV, Project PVP, Foundation MG. Immunization, Vaccines and Biologicals. 2011 Sep;2014:1–3.

[11] Nascimento EJM, George JK, Velasco M, Bonaparte MI, Zheng L, DiazGranados CA, et al. Development of an anti-dengue NS1 IgG ELISA to evaluate exposure to dengue virus. J Virol Methods. 2018;257:48–57. doi: 10.1016/j.jviromet.2018.03.007 29567514

[12] Bonaparte M, Huleatt J, Hodge S, Zheng L, Lustig Y, DiazGranados CA, et al. Evaluation of dengue serological tests available in Puerto Rico for identification of prior dengue infection for prevaccination screening. Diagn Microbiol Infect Dis. 2020;96(3):114918. doi: 10.1016/j.diagmicrobio.2019.114918 31839333

[13] DiazGranados CA, Bonaparte M, Wang H, Zhu M, Lustig Y, Schwartz E, et al. Accuracy and efficacy of pre-dengue vaccination screening for previous dengue infection with five commercially available immunoassays: Retrospective analysis of Phase III efficacy trials. Lancet Infect Dis. 2021. 10.1016/S1473-3099(20)30695-2 PMC9759790 33212068

[14] Hunsperger E, Peeling R, Gubler DJ, Ooi EE. Dengue pre-vaccination serology screening for the use of Dengvaxia®. J Travel Med. 2019;26(8):1–3. doi: 10.1093/jtm/taz092 31776549

[15] Perera S, John D, Senanayaka B. Cost effectiveness of dengue vaccination following pre-vaccination serological screening in Sri Lanka. Int J Technol Assess Health Care. 2019;35(6):427–35. doi: 10.1017/S0266462319000680 31625496

[16] Pearson CAB, Abbas KM, Clifford S, Flasche S, Hladish TJ. Serostatus testing and dengue vaccine cost-benefit thresholds. J R Soc Interface. 2019;16(157). doi: 10.1098/rsif.2019.0234 31431184 PMC6731500

[17] World Health Organization. Report of the first meeting of the WHO Diagnostic Technical Advisory Group for neglected tropical diseases. Geneva; 2019. p. 30–1. Available from: https://www.who.int/neglected_diseases/resources/9789240003590/en/.

[18] Hunsperger EA, Yoksan S, Buchy P, Nguyen VC, Sekaran SD, Enria DA, et al. Evaluation of commercially available diagnostic tests for the detection of dengue virus NS1 antigen and anti-dengue virus IgM antibody. PLoS Negl Trop Dis. 2014 Oct;8(10):e3171. doi: 10.1371/journal.pntd.0003171 . 25330157 PMC4199549

[19] Wilder-Smith A, Preet R, Renhorn KE, Ximenes RA, Rodrigues LC, Solomon T, et al. ZikaPLAN: Zika Preparedness Latin American Network. Glob Health Action. 2017;10(1):1398485. doi: 10.1080/16549716.2017.1398485 . 29235414 PMC7011980

